# Surface-grafted polyethylene glycol conformation impacts the transport of PEG-functionalized liposomes through a tumour extracellular matrix model†

**DOI:** 10.1039/c7ra13438j

**Published:** 2018-02-16

**Authors:** Hagar I. Labouta, M. Juliana Gomez-Garcia, Christopher D. Sarsons, Trinh Nguyen, Jacob Kennard, Wayne Ngo, Kaisha Terefe, Nicolas Iragorri, Patrick Lai, Kristina D. Rinker, David T. Cramb

**Affiliations:** Department of Chemistry, Faculty of Science, University of Calgary Canada dcramb@ucalgary.ca; Biomedical Engineering, University of Calgary Canada kdrinker@ucalgary.ca; Department of Pharmaceutics, Faculty of Pharmacy, Alexandria University Egypt; Health Technology Assessment Unit, Department of Community Health Sciences, Cumming School of Medicine, University of Calgary Canada; Department of Biological Sciences, University of Calgary Canada; Department of Physiology and Pharmacology, University of Calgary Canada; Centre for Bioengineering Research and Education, University of Calgary Canada; Department of Chemical and Petroleum Engineering, University of Calgary Canada

## Abstract

The effect of surface PEGylation on nanoparticle transport through an extracellular matrix (ECM) is an important determinant for tumor targeting success. Fluorescent stealth liposomes (base lipid DOPC) were prepared incorporating different proportions of PEG-grafted lipids (2.5, 5 and 10% of the total lipid content) for a series of PEG molecular weights (1000, 2000 and 5000 Da). The ECM was modelled using a collagen matrix. The kinetics of PEGylated liposome adhesion to and transport in collagen matrices were tracked using fluorescence correlation spectroscopy (FCS) and confocal microscopy, respectively. Generalized least square regressions were used to determine the temporal correlations between PEG molecular weight, surface density and conformation, and the liposome transport in a collagen hydrogel over 15 hours. PEG conformation determined the interaction of liposomes with the collagen hydrogel and their transport behaviour. Interestingly, liposomes with mushroom PEG conformation accumulated on the interface of the collagen hydrogel, creating a dense liposomal front with short diffusion distances into the hydrogels. On the other hand, liposomes with dense brush PEG conformation interacted to a lesser extent with the collagen hydrogel and diffused to longer distances. In conclusion, a better understanding of PEG surface coating as a modifier of transport in a model ECM matrix has resulted. This knowledge will improve design of future liposomal drug carrier systems.

## Abstract 2

The effect of surface PEGylation on nanoparticle transport through an extracellular matrix (ECM) is an important determinant for tumor targeting success.

## Graphical abstract

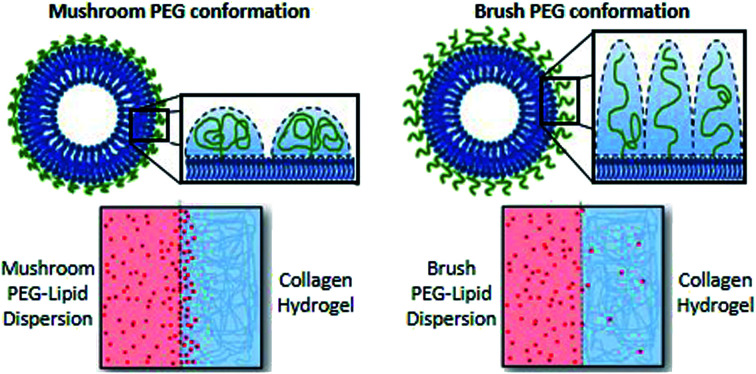

## Introduction

Understanding the relationship between nanoparticle design parameters and transport behaviour in the human body is critical to designing the next generation nanocarriers. The realization that nanoparticle behavior in biological systems is largely governed by interactions at the particle surface has led to a need for engineering the surface properties of nanoparticles.^1–4^ Functionalization of nanoparticles with polyethylene glycol (PEG) is a common technique to improve their stability in biological media.^5,6^ PEGylation also helps particles avoid clearance by the reticuloendothelial system, allowing for a prolonged circulation half-life. This works in part by manipulating the adhesion of blood serum proteins to the surface of the particles.^7^ PEG coatings reduce opsonization and thereby decrease the ability of phagocytes to hone-in on the particles.^8^ Because of the immune-modulating effects of PEG surface coatings, particles coated with PEG are often referred to as “stealth” particles.^9,10^

While deep tissue penetration is often a priority for nanoparticle drug delivery systems, especially those targeted to tumours,^11^ questions remain as to what, if any, effects these stealth coatings have on diffusion through a tumour-related extracellular matrix (ECM). Recently, the ECM has been identified as a substantial barrier for nanoparticle drug delivery agents, particularly in tumours.^12–15^ Fibrosis is a common feature of solid tumours, characterized by fibroblast recruitment,^16^ and consequent ECM deposition and remodeling.^17–19^ The resulting dense ECM impedes the penetration of nanoparticles in a size and charge dependent manner.^20,21^ Furthermore, collagen content has been identified as the primary contributor to the ECM's barrier effect.^22,23^

Recent work by our group, in collaboration with researchers at the University of Toronto, found that type I collagen is increasingly localized around tumour blood vessels as the tumours mature, thus increasing the obstacles faced by tumour penetrating nanoparticles.^20^ Physical, chemical and electrostatic interactions between the particles and collagen may impact the transport of particles through ECM.

Further complicating the matter is understanding how the variety of different PEG coatings commonly applied to nanoparticle drug delivery systems affects transport through dense media. Variables include: the molecular weight of the surface-grafted PEG, PEG surface density, and the terminal group of the PEG chains, along with less common variations such as the use of branched or bivalent forms of the polymer.^7,24–28^ Molecular weights typically range from 1–20 kDa,^24,25^ with larger PEG chains (≥5 kDa) demonstrating better mediation of protein absorption.^26^ There is less consistency in PEG surface density reporting, but published ranges include: 0–0.08 PEG chains nm^−2^,^7^ 0–20 wt% on the surface^27^ and 0–15 mol% on the surface.^28^

**Fig. 1:**
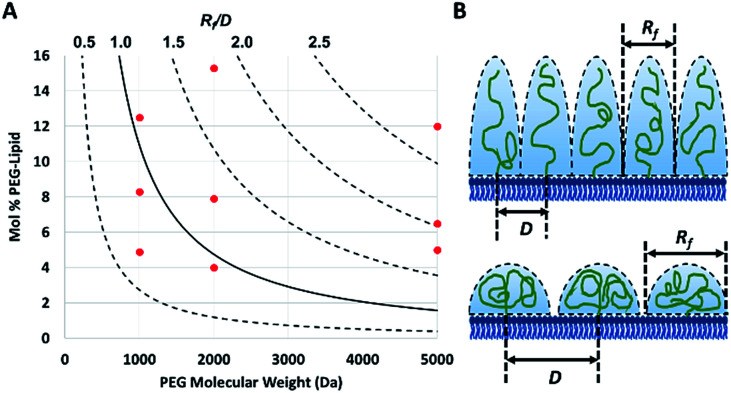
(A) The theoretical conformation of surface bound PEG for each of the nine PEGylated liposome formulations (red dots) plotted based on their measured fraction of PEG (with molecular weights of 1000, 2000 or 5000 Da). The calculated ratio (plotted as curved lines) of the Flory dimension (*R*_f_) over the average distance between two PEG attachment points (*D*) determines the conformation of a particle, with a *R*_f_/*D* of less than 1.0 representing mushroom conformation and greater than 1.0 and 2.0 representing brush and dense brush conformations, respectively. (B) The physical meaning of different *R*_f_/*D* values is illustrated (top: *R*_f_/*D* > 2.0 and bottom: *R*_f_/*D* < 1.0).

The PEG coil size (Flory dimension, *R*_f_), which is a function of the PEG molecular weight, and the PEG surface density determines whether the conformation of the PEG polymers on the surface of the particles is mushroom or brush.^29^ The mushroom conformation occurs when the average distance between the attachment points of two adjacent PEG chains (*D*) is greater than *R*_f_ of the polymer. As a result, each polymer chain interacts primarily with itself as opposed to interacting with neighboring polymer chains. In contrast, for brush conformation, PEG chains are grafted closer together, forcing the polymers chains to take on an elongated conformation, against their natural tendency to coil in upon themselves. Inter-chain interactions are dominant in the brush conformation. In theory, it is possible to predict the conformation of PEG for a PEGylated liposome formulation, as long as the PEG molecular weight and surface density are known.^29^ The calculated ratio of the Flory dimension to the average distance between adjacent PEG chains (*R*_f_/*D*) can be used as a reporter for PEG conformation: values below 1.0 indicate a mushroom regime, while those above 1.0 indicate brush (Fig. 1). As *R*_f_/*D* approaches zero, inter-chain interactions become more and more scarce and areas of bare particle between PEG chains begin to dominate the particle surface. On the other hand, *R*_f_/*D* values closer or larger than 2.0 represent denser brush configurations. Being able to predict the conformation of PEG on a particle surface can be quite important for particle design. PEG conformation significantly impact its behaviour in biological systems, as it has previously been shown to influence the cellular uptake efficiency,^30^ protein absorption,^7^ and biodistribution^31^ of nanoparticles.

The study presented herein addresses the effect of different PEG conformations on adhesion to and transport through collagen hydrogels. Collagen, as the major component of tumour ECM and the primary barrier to particle diffusion, was chosen to model tumour ECM *in vitro*. Liposomes were selected as model nanoparticles due to their facile functionalization and biomedical value.^32^ Ten different liposome formulations were prepared, each with a unique PEG surface coating, covering a range of conformations (represented by *R*_f_/*D*). The liposome preparation protocols allowed the nanoparticle size to be conserved among all formulations, resulting in the isolation of PEG conformation as the sole experimental variable.

## Experimental

### Preparation of fluorescent PEG-grafted liposomes

Lipid film hydration technique was used to obtain monodispersed unilamellar vesicles, following a previously published method after modification.^33^ Different fluorescent liposomal formulations were prepared based on 1,2-dioleoyl-*sn*-glycero-3-phosphocholine (DOPC) as the main lipid component with different proportions of PEG-grafted 2-dioleoyl-*sn*-glycero-3-phosphoethanolamine (DOPE) (2.5, 5 and 10% of the total lipid content) having different PEG molecular weights (1000, 2000 and 5000 Da). Liposomes were made fluorescent using a rhodamine labelled-DOPE “Lipid-rB” at a DOPC to Lipid-rB molar ratio of 200 : 1. All lipids were purchased from Avanti Polar Lipids, Inc. (Alabaster, Alabama, USA).

Lipids were dissolved in 5 ml chloroform (Sigma, St. Louis, Missouri) at different molar concentrations and vortexed (VWR Analog Vortexer Mixer, Radnor, Pennsylvania, United States) at 300 rpm for 5 minutes then dried under nitrogen. The formed thin lipid film was hydrated by adding 5 ml of water while vortexing at 300 rpm for 45 minutes to form liposomes. Prepared multilameller liposomes were extruded (Miniextruder from Avanti Polar lipids Inc. Alabaster, Alabama, Unites States) through a 200 nm followed by a 100 nm polycarbonate membranes (Nucleopore track-etch membranes by Whatman nucleopore, Pittsburg, Pennsylvania, USA), 25 times each. Liposomes were then protected from light and stored at 4 °C prior to characterization and further use.

### Characterization of liposomes colloidal properties

Liposomes were characterized for mean diameter and polydispersity index (PDI) using dynamic light scattering (DLS) and zetapotential using electrophoretic light scattering (ELS; ZetaSizer Nano DTS 1060, Malvern Instruments Ltd., Malvern, Worcestershire, UK) without further dilution at a temperature of 25 °C, a backscatter measurement angle of 173°, and an incident laser wavelength of 633 nm. Colloidal stability of liposomes at storage conditions was tracked over a period of 6 days. Measurements were performed in triplicates and values were reported as mean ± standard deviation.

### Determination of liposomes surface-grafted PEG density

The surface PEG density on liposomes was determined indirectly by quantifying the liposomal PEG-grafted lipid content by ^1^H NMR using Bruker AVANCE III RDQ400 NMR instrument with BBFO probe^34^ relative to the total phospholipid concentrations determined by an inorganic phosphate analysis,^35^*i.e.* mole fraction PEG-lipid. Liposomes with various PEG content were dried using Bligh–Dyer method^36^ then dissolved in CDCL_3_ using 0.2% v/v dimethyl sulphoxide (DMSO) as an internal standard. Relaxation time was set at 2 s and the number of scan was 64 scans. The peak chemical shifts were recorded at 2.64 ppm and 3.67 ppm for DMSO and PEG(OCH), respectively. The concentration of each of the PEG-grafted lipids with different PEG molecular weights (1000, 2000 and 5000 Da) was determined in reference to a calibration curve constructed from serial concentrations of the PEG-grafted lipid being measured in CDCl_3_ by ^1^H NMR using DMSO as an internal standard at the same concentration. Samples were measured in triplicates and data are presented as the mean ± standard deviation.

An inorganic phosphate assay was used to obtain the phospholipid concentrations.^35^ This assay is based on the reaction of phosphate with molybdenum to form the phosphomolybdate complex, which can be quantified by measuring the optical density at 820 nm. 50 μL were taken from each of the liposome samples and added to 10 × 70 mm glass test tubes. Subsequently, 30 μL of a 10% w/v MgNO_3_·4H_2_O in 95% ethanol was added followed by gently heating over a Bunsen burner to ash the samples. To cleave the phosphate from the lipid, 300 μL of 0.5 M HCl were added to the tubes followed by boiling for 15 minutes in a water bath. Then 700 μL of a 6 : 1 (0.42% w/v (NH_4_)_6_Mo_7_O_24_·4H_2_O in 0.5 M H_2_SO_4_: 10% w/v ascorbic acid_(aq)_) were added. The phosphomolybdate complex was formed after incubation at 37 °C for 1 hour and then the absorbance was measured using a UV-vis Spectrophotometer (Shimadzu UV-1700, Mandel Canada). Sample concentrations were calculated based on the absorbance of a 2 mM phosphate standard.

### Theoretical determination of surface PEG conformation

Surface PEG conformation (mushroom or brush conformation) was determined based on the calculated ratio of the Flory dimension (*R*_f_) to the average distance between adjacent PEG chains (*D*).^29^ PEG Flory dimension (eqn (1)), and the distance between surface grafted PEG chains (eqn (2)) were calculated using the equations by Kenworthy *et al.*^29^ where *a* is PEG monomer size in Å (previously reported as 3.8 Å (ref. 37)), *N* is the degree of polymerization, *A* is the PEG area per lipid molecule in the bilayer (previously reported as 67 Å^2^ (ref. 38)) and *M* is the mole fraction of PEG lipid determined experimentally as described above

1 *R*_f_ = *aN*^3/5^

2
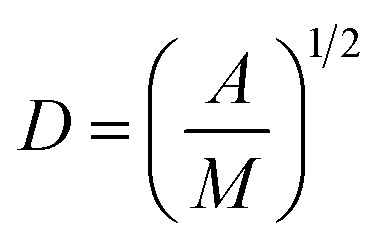


Calculated *R*_f_/*D* values below 1.0 indicate a mushroom regime, while those above 1.0 indicate brush.

### Measuring liposome migration from dispersion to hydrogel

#### Collagen hydrogel preparation and setting-up the adhesion experiment

**Fig. 2:**
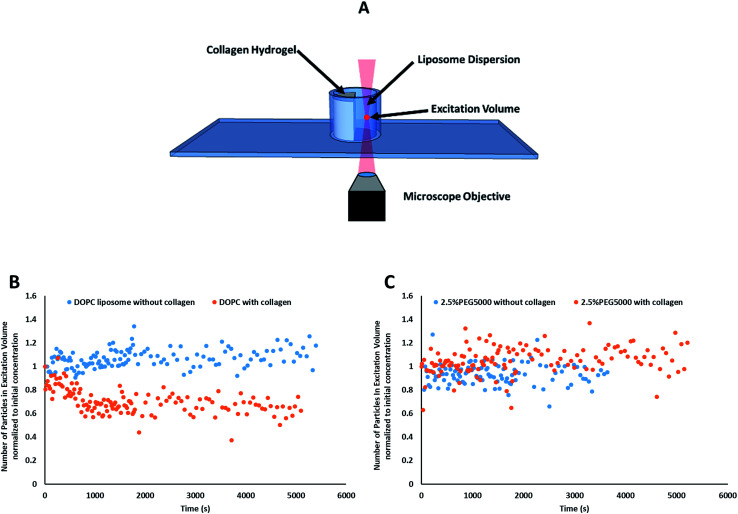
(A) A schematic depicting microscopic examination of liposomes adhesion to collagen hydrogel using fluorescence correlation spectroscopy (FCS). Control experiments were conducted in absence of collagen hydrogel. (B) Representative kinetics results obtained for FCS adhesion studies, where adhesion was observed for control DOPC liposomal formulation. Plot of the average number of particles (*N*) in the focal volume normalized to initial concentration as a function of time in the presence (red circles) and absence of collagen (black squares). (C) Data from “2.5% Lipid-PEG-5k” did not show a decrease in liposome concentration over time suggesting those liposomes are not adhering to the collagen hydrogels in measurable amounts.

Collagen hydrogel was prepared by mixing 150 μL of a 5 mg mL^−1^ collagen Type I solution (Rat Tail Bornstein and Traub Type I Sigma Aldrich® (Sigma Type VII) Powder) in 0.1 M acetic acid, Dulbecco's phosphate buffered saline (DPBS) (Life Technologies®), 120 μL of 0.625 M sodium hydroxide solution. The final concentration of collagen in the hydrogel was 2.5 mg mL^−1^. All solutions except for collagen were cooled in an ice bath prior to mixing to slow down the hydrogelation process for an optimal homogenous hydrogel matrix that is formed on incubation at 37 °C for 3 h in custom-made quartz chamber slides (Fig. 2A) covered with a glass cover slip at 37 °C for 3 h. The hydrogel surface was then gently rinsed five times with 400 μL of distilled water. Prepared hydrogels were then hydrated for a minimum of 2 h with 400 μL of distilled water.

#### Tracking liposome concentrations by fluorescence correlation spectroscopy (FCS)

The optical set-up as well as the equations used for data analysis were earlier developed by our group.^39–41^ In short, an upright Zeiss Axiovert 200 (Mississauga, ON, Canada) fluorescent microscope with a 40× water immersion objective lens (1.2 NA and 0.8 mm working distance) was used for collecting FCS data. Two photon excitation (TPE) was achieved using a mode-lock Ti: Sapphire, 100 fs pulsed laser (Tsunami, Spectra Physics, Palo Alto, CA) operating at 82 MHz and 780 nm with a power of 60 mW at the overfilled back aperture of the microscope objective. This set-up resulted in an estimated TPE volume of *ca.* 2.50 × 10^−15^ L. Fluorescence emission from the flourophores was collected back through the same objective lens and reflected off a dichroic optic (Chroma 100DCSPXr, Rockingham, VT), which separated the emitted fluorescence from the excitation source. The fluorescence was then directed through a band-pass filter D605/40 m (Rockingham, VT) and detected by an avalanche photodiode detector (Photon Counting Module SPCM CD2882; Perkin-Elmer, Vaudreuil, QC, Canada). The signal was then recorded using a Correlator Board (ALV5000/E, Langen, Germany), and the resulting decay curves were further analyzed.

Prior to data collection, a volume of 350 μL liposomal dispersion was gently pipetted into the adhesion chamber slide, zero time (Fig. 2B). FCS was used to track the liposomes concentration at a focal point in the liposomes dispersion away from the collagen surface for 90 minutes (starting from 10 seconds after liposomal addition to the well) using the optical settings mentioned above *versus* negative control trial conducted in absence of collagen (zero time).

#### FCS data processing

Single autocorrelation data was processed using Origin Pro 7.0 Data Fitting software (OriginLab Co.). The autocorrelations were fitted to using eqn (3) (ref. 42 and 43) *via* a Levenberg–Marquardt algorithm; where *G*(0) is the correlation amplitude, *D*_H_ is the hydrodynamic diffusion coefficient, *τ* is the lag time, *r* is the radius of the laser beam, *z*_0_ is the depth of the focal volume, the excitation volume was 2.5 × 10^−9^ L at 15 mW laser power; *r*^2^ and *z*_0_ were 1.4 × 10^−13^ m^2^ and 9.2 × 10^−6^ m, respectively.3
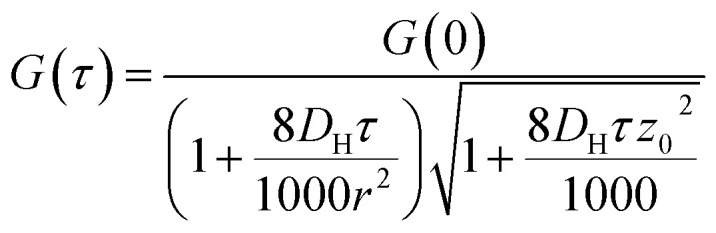


*G* values and the diffusion coefficients were extracted from the autocorrelation data. Particle numbers (*N*) were determined from *G* values using eqn (4).^44^4
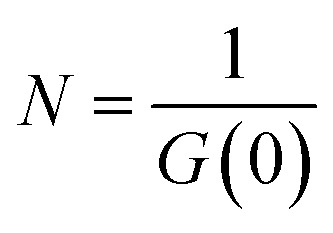


*N* values were plotted against the recorded time of each run to give a rate graph. Data points up to the first 2000 s were used in determination of rate constants. Rate constants (*k*_loss_ and *k*′_loss_) were determined by fitting the curves with a bi-exponential decay equation (eqn (5)), where *N*(0) and *N*(*t*) represent the concentration of the species being measured at time zero and time *t* and *c*, *a* and *b* are the hybrid constants bi-exponential decay equation.

5 *N*(t) = *N*(0)[*c* + *ae*^−*k*_loss_*t*^ + *be*^−*k*′_loss_*t*^]

We used a bi-exponential decay, because the initial particle adhesion appears to saturate (to a degree) leading to a slower adhesion process at longer times. The evidence for this comes from the gel uptake data presented below and a slow long tail in the FCS-measured loss kinetics. In fact, when *k*′_loss_ is very small compared to the measurement time, the loss kinetics can appear to be zero order.

#### Statistical analysis

Two-Way ANOVA with post-hoc Tukey testing was performed on the rate constant data using PEG molecular weight and molar percentage of PEG as variables. One-Way ANOVA was also performed on the rate constant data, using the types of liposomes as the variable.

### Tracking liposome transport in collagen hydrogels

#### Building custom transport chamber

A transport chamber made of polyvinyl chloride acetate (PVCA) covered with a glass microscopical cover slip (Fisherbrand®) was built in a 35 mm Petri Dish (MatTek Corporation®). A schematic diagram of the transport chamber is shown in Fig. 4A.

**Fig. 3:**
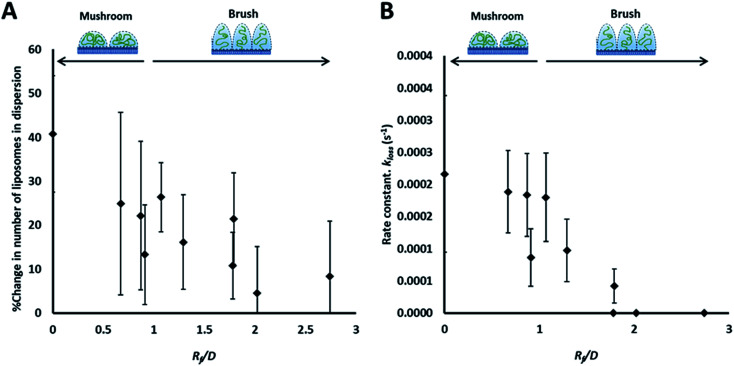
Summary of the results of the adhesion study of the prepared liposomes to collagen hydrogel using fluorescence correlation spectroscopy, showing the percentage change in liposomes concentration (fluorescent events) in the focal volume after 90 min (A) and the rate constants of liposomes disappearance from the dispersion (*k*_loss_) obtained by fitting kinetics plots (examples shown in Fig. 2B and C) with an exponential decay function (eqn (5)) (B).

**Fig. 4:**
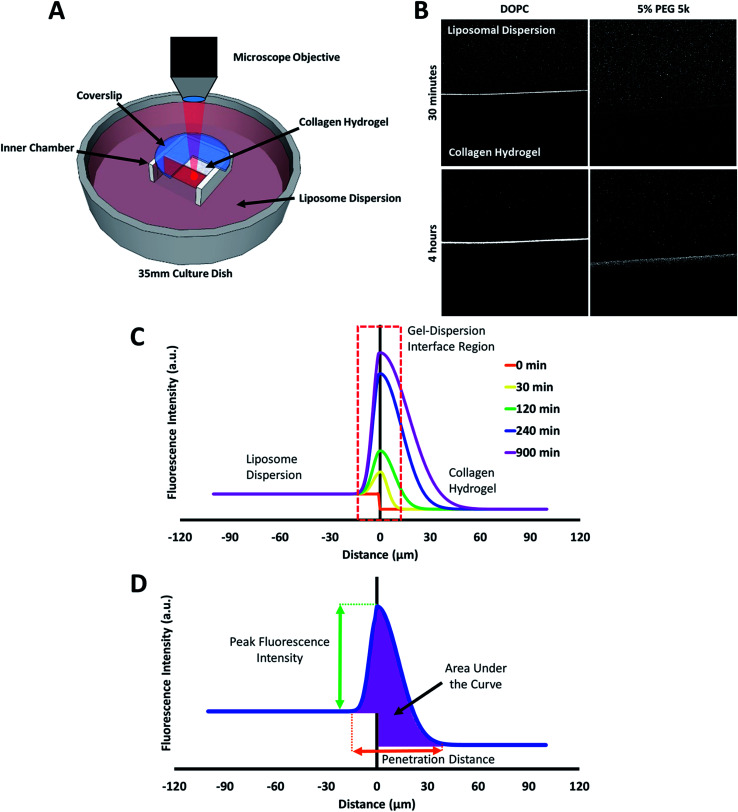
(A) Schematic diagram of the custom-made transport chamber used to observe the transport of fluorescent liposomes into, and through a collagen hydrogel. (B) Representative confocal images of DOPC and 5% PEG 5k liposome distribution in collagen hydrogels after 30 minutes and 4 hours. (C) A mock fluorescence line analysis curve highlighting the key regions observed in the liposome transport experiments. (D) A mock fluorescence line analysis curve and associated parameters for characterizing liposomes transport through collagen hydrogel.

#### Preparation of collagen hydrogel and setting-up the transport experiment

Collagen solution was similarly prepared as described in the adhesion experiments and allowed to hydrogel on incubation at 37 °C for 3 h in the inner compartment of transport chamber as shown in Fig. 4A. Following incubation, the prepared collagen hydrogel in the inner compartment was hydrated with 6 mL of distilled water pipetted to the outer chamber for a period of 2 h away from light, to prevent hydrogel crosslinking. Prior to imaging, a volume of 75 μL liposomal dispersion was added to the water in the outer chamber, zero time.

#### Tracking transport of liposomes through collagen hydrogel using confocal imaging

An upright Olympus® Confocal Microscope FV1000 with an UPLFLN 10× Olympus® water immersion objective lens was used to image the interface between the collagen hydrogel and the surrounding liposomal dispersion for a period of 15 h using a Rhodamine Red-X (R-RX) and TD1 Detection channel setting. Parafilm was used to cover the regions of the transport chamber exposed to air, limiting the evaporation of water over the period of imaging. Images were sequestered at the allotted time points (0, 5, 10, 20, and 30 minutes) using a Rhodamine Red-X detection channel setting of 700 V. The gain on the detection channel was then lowered to 550 V, to minimize the possibility of having saturated signals due to liposomes for the following time points (every 30 min up to 15 h).

#### Image analysis

Image J freeware, version 1.49t was used to measure the average fluorescence intensity along a line perpendicular to the collagen hydrogel-liposome dispersion interface. Fluorescence line analysis was performed 10 times per time point. For each line analysis data set, plotting the average fluorescence intensity against the distance in μm from the interface resulted in a bell-shaped curve. The arbitrary line was adjusted so that the “zero point” lies at the center of the interface between the hydrogel and the liposome dispersion, whereas negative and positive distance values are allocated for distances within the liposome dispersion and the collagen hydrogel, respectively. From these generated graphs, three parameters (peak fluorescence intensity, width of peak fluorescence intensity and area under the curve) were used to characterize the different liposomes transport into the hydrogel. These parameters are outlined in Fig. 4C. Additionally, rates of accumulation of liposomes in the collagen hydrogels were calculated by plotting the measured AUC *versus* time and fitting the data to a zero order rate equation (eqn (6)), where [TAUC] is the total fluorescence intensity of the liposomes, *k*_a_ is the rate constant, and *t* is time.

6 [TAUC] = [TAUC]_0_ + *k*_a_*t*

The order of the rate equation was determined based on the trend found between AUC *versus* time (ESI Fig. 1A†). Linear regressions were calculated for every formulation to determine if a linear trend could best represent the relationship between these variables. Average *R*-squared values of the linear model fit for each liposomal preparation are shown in ESI Table 1.†

#### Statistical analysis

Generalized least square regressions were used to estimate the correlation coefficients between the measured transport parameters, and either of PEG molecular weight, surface density, conformation, and zeta-potential. The linear correlation between each transport parameter and time (within variability) was also estimated except for the rate constant of liposomal accumulation in collagen (*k*). Therefore, for *k*, ordinary least square linear regressions were used to estimate correlation coefficients. The variance inflation factor (vif) was estimated to avoid potential collinearity between regressors. A significance level of 0.05 was established. Statistical analyses were conducted in Stata 14 (StataCorp LLC).

## Results and discussion

Liposomes were used as model particles to study the effect of PEG surface functionalization on the transport properties of nanoparticles in ECM. Reproducible monodisperse particles were needed to ensure surface functionalization was the only property varied between formulations. The extruded liposomes prepared for this study had a mean hydrodynamic diameter ranging from 81 to 94 nm, a zeta potential ranging between −16.5 and −30 mV (Table 1), and were found stable in terms of colloidal properties for at least 6 days. As shown in Table 1, there was no significant variability in diameter between the formulations. This is similar to earlier results by Sriwongsitanont and Ueno,^45^ where increasing PEG surface density up to 10% did not significantly affect the liposome size. Earlier however, it was stated by Kenworthy *et al.*^29^ that as PEG loading increases, the PEG strands will be forced closer together on the surface of the liposome, increasing the lateral repulsive forces between PEG molecules. This repulsion causes greater curvature, and therefore smaller liposomes tend to be energetically favorable at high PEG concentrations, and eventually start to form micelles at very high concentrations. However, results obtained in our study did not show an effect of the PEG surface density on the vesicle size. This discrepancy can be explained by the use of extrusion to prepare monodispersed liposomes, where all liposome preparations were forced through a 100 nm membrane masking any effects PEG had on their diameters. Similarly, being non-ionic, varying both the molecular weight and surface density of PEG, did not have an effect on the zeta potential of the prepared liposomes as shown earlier.^46^ The consistency between particles of different formulations allowed us to isolate the PEG molecular weight and surface density – and ultimately conformation – as variables. Previous work has shown that both particle size and surface charge affect particle transport in ECM,^20,21^ highlighting the importance of controlling these parameters to precisely measure the effect of PEG on particle interactions with the collagen hydrogels.

**Table 1:** Composition of the prepared liposomes and their characterized properties (mean hydrodynamic diameter, polydispersity index (PdI) and zeta potential)

Formulation code	Concentration of individual lipids^a^, M					Measured PEG-lipid content^g^, mol%	*R*_f_/*D*	Hydrodynamic diameter, nm	PdI	Zeta potential, mV
	DOPC^b^	Lipid-PEG-1k^c^	Lipid-PEG-2k^d^	Lipid-PEG-5k^e^	Lipid-rB^f^					
Control	1 × 10^−4^	—	—	—	5.0 × 10^−7^	—	81.0 ± 6.9	0.13 ± 0.03	−14.1 ± 4.9	
2.5% lipid-PEG-1k	9.7 × 10^−5^	2.5 × 10^−6^	—	—		4.9 ± 0.2	0.67	87.4 ± 4.7	0.09 ± 0.03	−30.1 ± 3.2
5% lipid-PEG-1k	9.5 × 10^−5^	4.9 × 10^−6^	—	—		8.3 ± 0.1	0.87	87.3 ± 6.1	0.13 ± 0.04	−20.2 ± 4.8
10% lipid-PEG 1k	9.0 × 10^−5^	10.0 × 10^−6^	—	—		12.5 ± 0.2	1.07	84.7 ± 9.4	0.13 ± 0.04	−17.7 ± 4.0
2.5% lipid-PEG 2k	9.7 × 10^−5^	—	2.5 × 10^−6^	—		4.0 ± 0.3	0.91	87.4 ± 6.4	0.09 ± 0.03	−21.5 ± 2.7
5% lipid-PEG 2k	9.5 × 10^−5^	—	4.9 × 10^−6^	—		7.9 ± 0.4	1.29	89.3 ± 5.1	0.09 ± 0.03	−21.6 ± 4.9
10% lipid-PEG 2k	9.0 × 10^−5^	—	10.0 × 10^−6^	—		15.3 ± 0.4	1.79	89.9 ± 8.1	0.08 ± 0.05	−18.1 ± 2.1
2.5% lipid-PEG 5k	9.7 × 10^−5^	—	—	2.5 × 10^−6^		5.0 ± 0.4	1.78	89.6 ± 5.2	0.13 ± 0.04	−16.7 ± 3.4
5% lipid-PEG 5k	9.5 × 10^−5^	—	—	4.6 × 10^−6^		6.5 ± 0.2	2.02	93.6 ± 4.1	0.10 ± 0.04	−17.0 ± 1.8
10% lipid-PEG 5k	9.0 × 10^−5^	—	—	9.2 × 10^−6^		12.0 ± 0.1	2.74	90.5 ± 6.9	0.10 ± 0.04	−16.5 ± 0.9

a The concentration of the individual lipid ingredients are the theoretical lipid concentrations.

b DOPC: 2-dioleoyl-*sn*-glycero-3-phosphocholine.

c Lipid-PEG-1k: 1,2-dioleoyl-*sn*-glycero-3-phosphoethanolamine-*N*-[methoxy(polyethylene glycol)-1000] (ammonium salt).

d Lipid-PEG-2k: 1,2-dioleoyl-*sn*-glycero-3-phosphoethanolamine-*N*-[methoxy(polyethylene glycol)-2000] (ammonium salt).

e Lipid-PEG-5k: 1,2-dioleoyl-*sn*-glycero-3-phosphoethanolamine-*N*-[methoxy(polyethylene glycol)-5000] (ammonium salt).

f Lipid-rB: 1,2-dioleoyl-*sn*-glycero-3-phosphoethanolamine-*N*-(lissamine rhodamine B sulfonyl).

g Measured lipid-PEG-1k, lipid-PEG-2k or lipid-PEG-5k using ^1^H NMR.

To verify the surface density of PEG matched the stoichiometric predictions, the actual concentration of PEG-grafted lipid in the liposomes was measured by quantitative ^1^H NMR (Table 1). Measurements were performed in triplicate for each PEGylated liposome preparation. While all of the liposome formulations contained roughly the anticipated PEG content, most formulations had higher than expected PEG content. It is not clear whether this apparent trend represents a physical, reproducible phenomenon, or rather is a coincidental occurrence. Differences in PEG content may be explainable by variations in the interactions between the PEG-lipids and the extrusion apparatus and/or different self-assembly equilibria reached by the different preparations. However, this phenomenon was not studied directly and any physical explanation is purely speculative. Although the PEG density did not always match stoichiometric predictions, the actual surface loading is still expected to yield liposomes with brush and mushroom PEG conformations, according to theoretical calculations^29^ (Fig. 1).

Particle activity at the interface between an aqueous and a hydrogel environment has previously been identified as an important driver of particle transport in ECM.^20^ A novel assay was developed to further study this activity. Liposome dispersions were added to custom-built quartz chamber slides (Fig. 2), both in the presence and absence of collagen hydrogels, and the concentration of liposomes in the dispersion was then tracked by FCS over a period of 90 minutes. In absence of collagen hydrogels, all liposome preparations were observed to have a stable liposome concentration, suggesting that aggregation and adhesion to the quartz chamber slide do not significantly take place. The control (pure DOPC) liposomes exhibited significant decreases in concentration when exposed to the collagen hydrogel (Fig. 2B). The same was also observed for the liposomes with 1 kDa and 2 kDa PEG surface coating (at all surface densities). The liposomes with 5 kDa PEG surface coatings however did not display any significant decrease, regardless of surface density (Fig. 2C). This suggests that the liposomes with 5 kDa PEG surface coatings are not adhering to the collagen hydrogels in measurable amounts. Although it seems unlikely that the 5 kDa PEG liposomes were entirely excluded from interactions with the hydrogels, the interactions were likely reduced to a point where they were masked by noise in the data. Additionally, the slower *k*′_loss_ kinetics values had large standard deviation owing to the large background of free particles. The χ^2^ (goodness of the fits) did improve using this parameter, but the longer time adhesion process would be better measured from the gel side of the process.

The rate of liposome disappearance for the dispersions was determined by fitting the FCS results with eqn (3), representing first order association between the liposomes and the hydrogel (Fig. 3A). The rate constant, *k*_loss_, was calculated for each experimental run. The mean *k*_loss_ for each liposome formulation is displayed in Fig. 3B. Varying the molecular weight of PEG chains at the surface of liposomes had a significant effect on the rate of liposome disappearance from the dispersions. Adhesion rate constants decreased as PEG molecular weight increased. The 5k-PEG liposome data was not able to be fitted using eqn (3) as these experiments resulted in no significant loss over the observation period. The surface density of PEG did not affect the rate of adhesion as the rate constants for each PEG molecular weight level remained statistically indistinguishable regardless of changes in PEG loading. PEG conformation (Fig. 3) was found to be a better predictor of differences in liposome behaviour than either molecular weight, or surface density (ESI Fig. 2†).

The behaviour of the particles in contact with the hydrogels was monitored in a separate experiment. Liposomes in dispersion were allowed to traverse a collagen hydrogel prepared in the inner compartment of a custom-built transport chamber (as illustrated in Fig. 4A and B). The movement of the fluorescently-labeled liposomes was then tracked by confocal microscopy over a period of 15 hours. All of the liposome preparations followed the same general behavior; an illustration of this generalized behavior can be found in Fig. 4C. Within the first two hours, the liposomes began to accumulate along the interface between the liposome dispersion and the collagen hydrogel. The signal of liposomes in the interface region became much greater than that of the liposomes in the dispersion. This indicates that concentration-driven diffusion does not explain the transport phenomenon at work at the interface region.

A similar result was previously reported by our group using PEGylated gold nanoparticles in a similar assay.^20^ The fact that both sets of particles were PEGylated suggests PEG-collagen interactions may contribute to this behavior. PEG-collagen interactions have previously been reported in thin films by Sionkowska *et al.*^47^ The same behavior was also observed in the uncoated liposomes used in this study, meaning some other interactions must be responsible for at least some of this effect. Both sets of particles were measured to have slightly negative zeta potentials, hinting at the possibility of electrostatic interactions with weakly positive collagen fibers.^48^

**Fig. 5:**
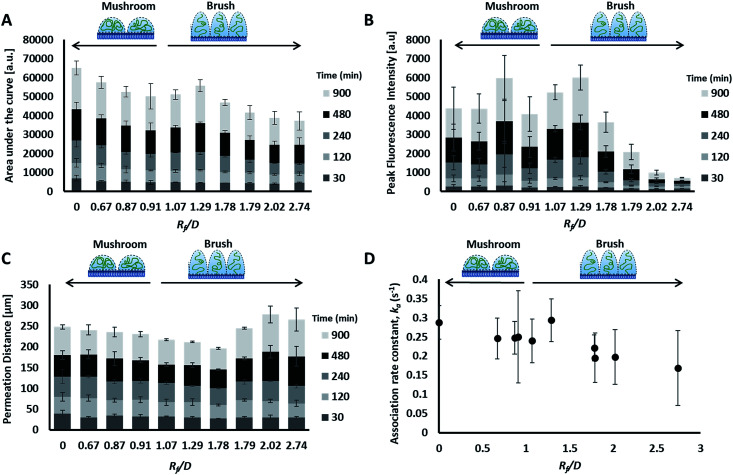
Curve characterization data for the transport of the prepared liposomes through collagen hydrogels. Distance *vs.* fluorescence intensity curves through time (30 to 900 minutes), were characterized by four metrics: area under the curve (AUC) (A), peak fluorescence intensity (B), permeation distance (C) and rate constant of accumulation (D). Legends include. Mean ± SD shown, *n* = 3–6.

The liposome transport within the dispersion-gel system was characterized by four metrics (Fig. 4D). The first metric aimed to quantify the total number of vesicles associated with the collagen hydrogel, by measuring the area under the curve (AUC) of the fluorescence intensity *vs.* distance plots. The second metric of interest is the peak fluorescence intensity, which occurred at the dispersion-gel interface in all cases, and represents the number of liposomes adhering to the interface. The third metric measures the total distance of elevated fluorescence; this metric defines the maximum penetration distance into the collagen hydrogel. The fourth metric fits the AUC as a function of time with a zero order rate equation, being the simplest interpretation of the data (average rate constants of liposome association with the collagen gels, *k*_a_). Each of the four metrics provides some unique information about the behaviour of the liposomes in our system. Changes in both PEG molecular weight and surface density yielded statistically significant differences in the liposomes behavior (Fig. 5), suggesting PEG conformation, expressed as *R*_f_/*D*, is an important driver of particle transport in collagen hydrogels.

Preparations with surface-grafted PEG in the mushroom and sparse brush conformation, *R*_f_/*D* < 1.5, exhibited higher accumulation of liposomes in the hydrogel (AUC) and increased association at the interface (peak fluorescence intensity), but shorter penetration distances (PD) (Fig. 5). The behaviour of these particles was similar to the control particles. As PEG molecular weights and surface densities were increased, through the sparse brush range and into a dense brush conformation, the transport behaviour was altered; including a falling off of peak fluorescence intensity (PFI) and AUC, accompanied by a slight increase in PD. However, PFI increase was not statistically significant from the other formulations including DOPC control particles, despite previous work suggests that PEGylation of nanoparticles does increase their mobility in ECM.^49,50^ It is noteworthy to mention that these altered behaviours of particles are not attributed to a difference in their mobility in water at the collagen interface; no significant differences were observed between their diffusion coefficients in water (refer to the ESI Fig. 3†).

**Fig. 6:**
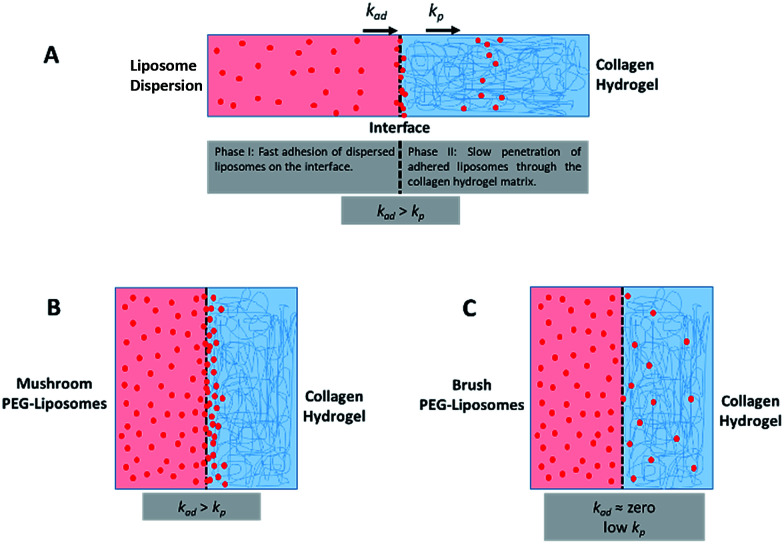
(A) A schematic representation of the different phases and rates of interactions (adhesion and penetration) of liposomes with the collagen hydrogel whereas adhesion rate constant (*k*_ad_) is higher than penetration rate constant (*k*_p_). (B) This is the case for mushroom PEG conformation liposomes in which adhesion predominates a more difficult step which is a penetration through the collagen matrix. (C) Brush PEG conformation liposomes do not adhere on the interface and penetrates the hydrogel at a low rate.

The fourth metric characterizing liposome transport within the dispersion-gel system is the association rate constant, *k*_a_. We observed an overall decrease in average association rate constants with the collagen gels (*k*_a_) with the increase of *R*_f_/*D* (Fig. 5). Based on AUC, PFI and PD results, we assume that a rate constant explains different phenomena for the different preparations (Fig. 6). Liposomes with surface-grafted PEG in the mushroom and sparse brush conformation accumulate the most on the interface with short PD; *k*_a_ stands for adhesion rate constant (*k*_ad_). On the other hand, the predominant phenomenon in case of liposomes with PEG in the dense brush conformation is penetration, *i.e. k* stands for penetration rate constant (*k*_p_). Assuming that adhesion at the interface occurs faster than penetration through a hydrogel matrix (*k*_ad_ > *k*_p_), this could explain the overall decrease rates with *R*_f_/*D*.

A generalized linear model was used to estimate if PEG molecular weight, surface density, *R*_f_/*D*, or zeta potential correlated with the transport measurements selected to describe the movement of liposomes into the collagen hydrogel (*i.e.* AUC, PFI, PD, and *k*_a_). Multiple regressions were conducted to estimate the correlation coefficients across time. Additionally, overall *R*-squared values and regression-specific *p*-values were obtained to evaluate the measure of fit for each comparison and the statistical significance of each coefficient, respectively. Results are summarized in Table 2, where dependent variable coefficient represents the magnitude of correlation between the transport parameter being evaluated and the formulation parameter (*i.e.* independent variable) and the time coefficient represents the change of the dependent variable in time.

**Table 2:** Summary of the correlations between different parameters characterizing the transport behaviour of liposomes in collagen hydrogels, with different metrics^a^ characterizing the PEG coatings of those liposomes

Independent variable	Dependent variable	Overall *R*^2^ for model	Dependent variable coefficient	Time coefficient^b^	*p*-Value dependent variable coefficient^c^
Molecular weight	AUC	0.9133	−0.749	14.081	<0.001
	PFI	0.6663	−0.143	1.4543	0.004
	PD	0.7282	0.0005	0.0399	0.563
	*k*_a_	0.1582	−9 × 10^−14^	NA	<0.001
Surface density	AUC	0.8855	−315.2	14.081	0.015
	PFI	0.513	−36.15	1.4543	0.3
	PD	0.7271	0.235	0.0399	0.62
	*k*_a_	0.1179	−4.07 × 10^−1^	NA	<0.001
Conformation (*R*_f_/*D*)	AUC	0.9391	−2088	14.081	<0.001
	PFI	0.6753	−356.1	1.4543	0.002
	PD	0.7315	1.697	0.03989	0.427
	*k*_a_	0.2205	−2.5703	NA	<0.001
Zeta potential	AUC	0.8455	−121.1	14.081	0.374
	PFI	0.5391	−29.27	1.4543	0.317
	PD	0.7253	0.121698	0.039889	0.76
	*k*_a_	0.0262	−0.1469	NA	0.028

a Area under the curve (AUC), peak fluorescence intensity (PFI), permeation distance (PD), association rate constant (*k*_a_).

b All *p*-values for time coefficients are <0.001.

c Non-significant values represent dependent variable coefficients that are statistically equal to zero, *i.e.* they are not affected the independent variable.

Molecular weight and surface density both correlated with total liposome association to the collagen hydrogel, AUC, however, particle conformation had the largest magnitude coefficient, suggesting that small changes in *R*_f_/*D* can result in significant variations of particle accumulation. Zeta potential measurements did not register significant differences in charge-shielding by surface-grafted PEG between formulations (Table 1 and ESI Fig. 4†), therefore zeta potential did not influence liposome interaction to or transport through the collagen hydrogel (Table 2). The differences in liposome behaviour with changing PEG conformation could be alternatively explained by PEG's shielding effect of the hydrophobic interactions between phospholipid bilayer and collagen. The degree of hydrophobic-shielding by PEG is dependent on the conformation of PEG on the surface: with dense brush-conformation PEG coatings yielding the greatest hydrophobic-shielding and lower density mushroom-conformation PEG coating yielding less hydrophobic-shielding.^51^ Exhibiting less hydrophobic interaction with the collagen fibers could explain why the particles with denser PEG regimes displayed lower peak intensity, lower AUC and higher penetration distance.

A constant increase in all association metrics (AUC, PFI, and PD) was found as time progressed (ESI Fig. 1A–C†). The relatively high magnitude of the linear coefficient with a *p*-value <0.001 between time and total association suggests that liposomes penetrate into the collagen hydrogel even after more than 15 hours had passed, *i.e.* equilibrium was not reached for all liposomes at 15 hours (ESI Fig. 1A†). Similarly, PFI was also observed to increase with time (ESI Fig. 1B†). However, PD was found to deviate from a linear behaviour with time except for liposomes with dense brush PEG conformation which continue to penetrate the collagen hydrogel for longer distances (ESI Fig. 1C†). Nonetheless, dense brush particles were barely present inside the collagen hydrogel (lower AUC and PFI values – ESI Fig. 1A and B†). Overall, differences in liposome association and permeation between formulations seem to be accentuated with time. During the first two hours of exposure, PEG molecular weight, surface density and conformation did not have a clear effect on particle transport. After 4 hours of exposure, PEG conformation seems to have a more important role on the number of particles that interact with the collagen hydrogel.

## Conclusions

PEG conformation was found as the best descriptor of the transport of PEG-grafted liposomes through ECM. Liposomes with mushroom PEG conformation (*R*_f_/*D* < 1) interacted to a great extent with the interface of collagen hydrogel limiting penetration into the hydrogels for long distances, in a similar fashion to uncoated control liposomes. Higher values of *R*_f_/*D* resulted in a lower particle accumulation on the collagen hydrogel-liposomal dispersion interface and higher penetration distances into the hydrogel. Even though liposomes with dense brush PEG conformation had the highest penetration distances into the hydrogel, they were present in sparse amount on or in the collagen hydrogel after 15 hour.

Implications of our findings for designing drug delivery systems for cancer targeting are less straight forward. Less dense PEG regimes allow more particles to accumulate within the ECM, potentially increasing the dose in the tumour tissue. On the contrary, high dense PEG regimes allow particles to penetrate deeper into the tumour tissue but a higher particle dose might be needed to achieve therapeutic effect of the encapsulated anticancer drug. Although it seems there may be no simple one-formulation-is-best conclusion, we now have a better understanding of how surface functionalization can impact nanoparticle transport inside the human body.

## Conflicts of interest

There are no conflicts to declare.

## Supplementary Material

RA-008-C7RA13438J-s001
