# Supplementary material for: Surface-grafted polyethylene glycol conformation impacts the transport of PEG-functionalized liposomes through a tumour extracellular matrix model
Source: RSC Adv. 2018 Feb 16;8(14):7697–708. doi: 10.1039/c7ra13438j (PMC9078461; doi:10.1039/c7ra13438j)
Supplement: RA-008-C7RA13438J-s001 [file RA-008-C7RA13438J-s001.pdf]

Electronic Supplementary Material (ESI) for RSC Advances.
This journal is © The Royal Society of Chemistry 2018

Supplementary Information

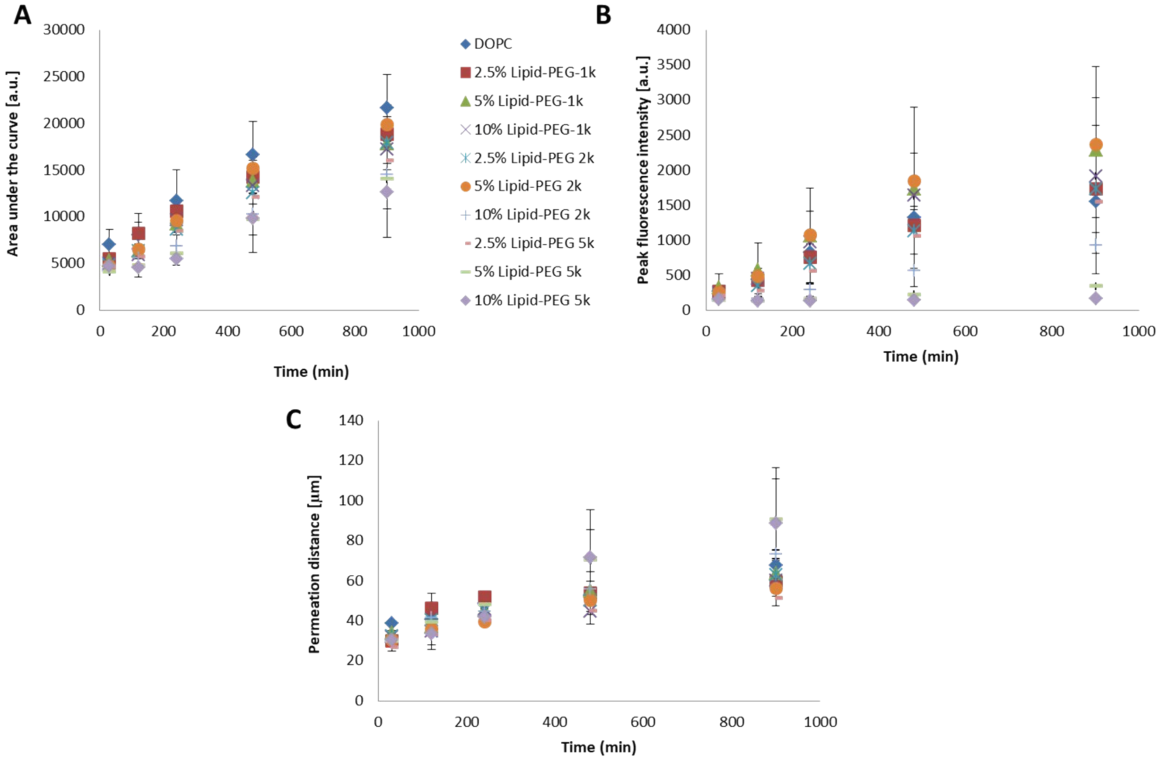

Supplementary Figure 1. Liposome association with the collagen matrix over time. (A) Area under the curve represents the total association of particles to the collagen gel. (B) Peak fluorescence intensity denotes the accumulation of particles at the interface of water and collagen matrix. (C) Permeation distance is the maximum distance at which liposomes where found in the collagen gel. Legend in the middle of the top row apply to all three plots.

A.

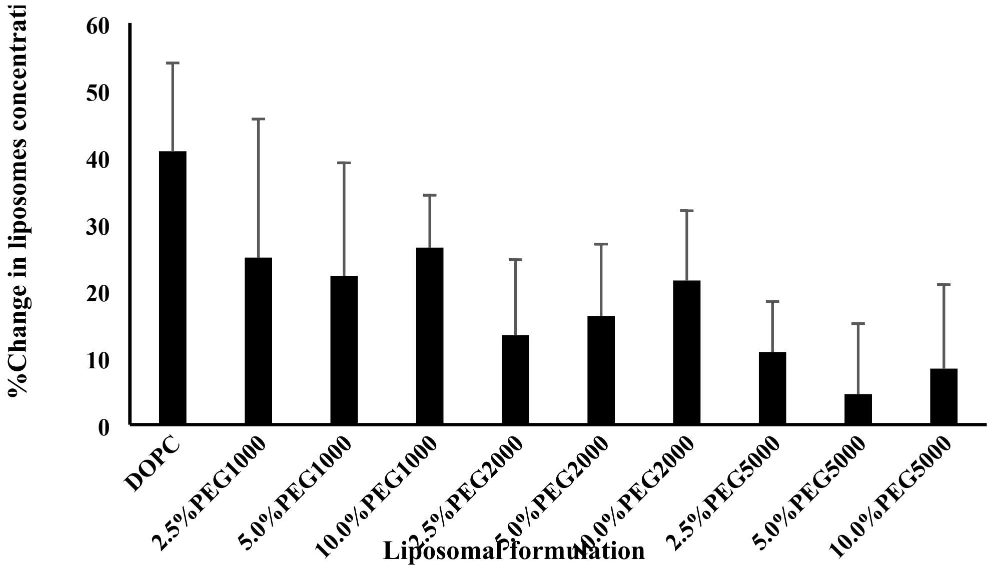

B.

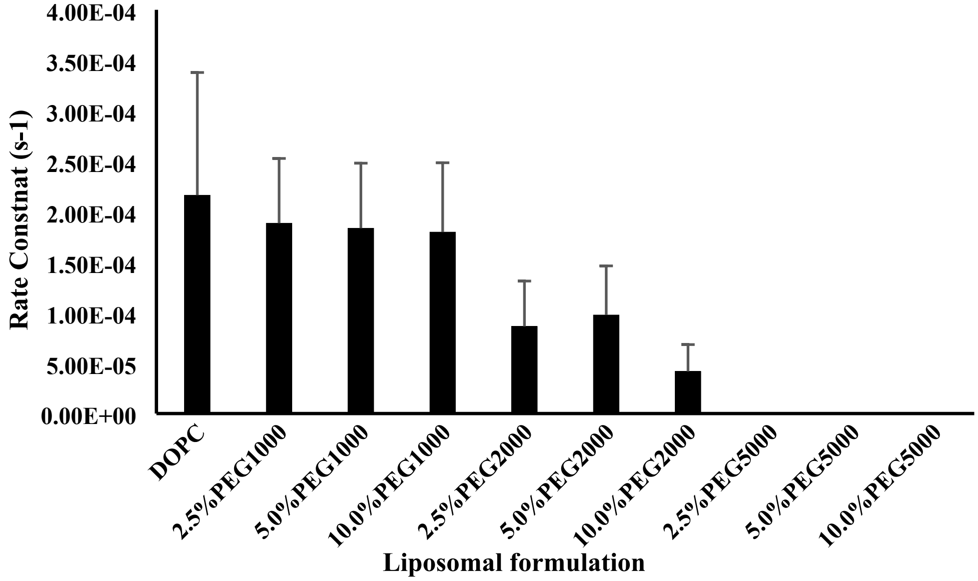

Supplementary Figure 2: Summary of the results of the adhesion study of the prepared liposomes to collagen gel using fluorescence correlation spectroscopy, showing the percentage change in liposomes concentration (fluorescent events) in the focal volume after 90min (A) and the adhesion rate constants obtained by fitting kinetics plots with an exponential decay function (Equation 3 in the main manuscript) (B).

Fluorescence correlation spectroscopy measurements were used to determine the diffusion coefficients for each liposomal formulation, averaged from the first five runs of each replicate. The diffusion coefficients are reported as an average of five independent replicates; SEM bars are included.

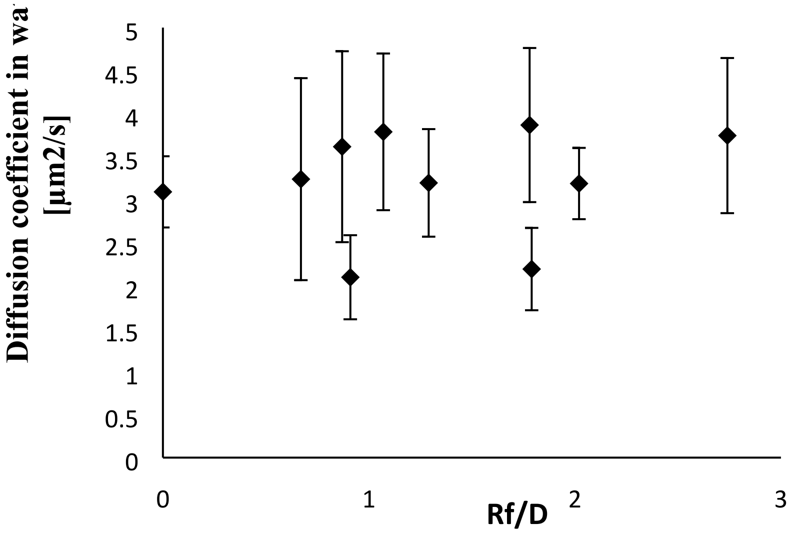

Supplementary Figure 3: Diffusion coefficient of liposomes in water calculated using Fluorescence correlation spectroscopy. Error bars for FCS denote SE for n = 5.

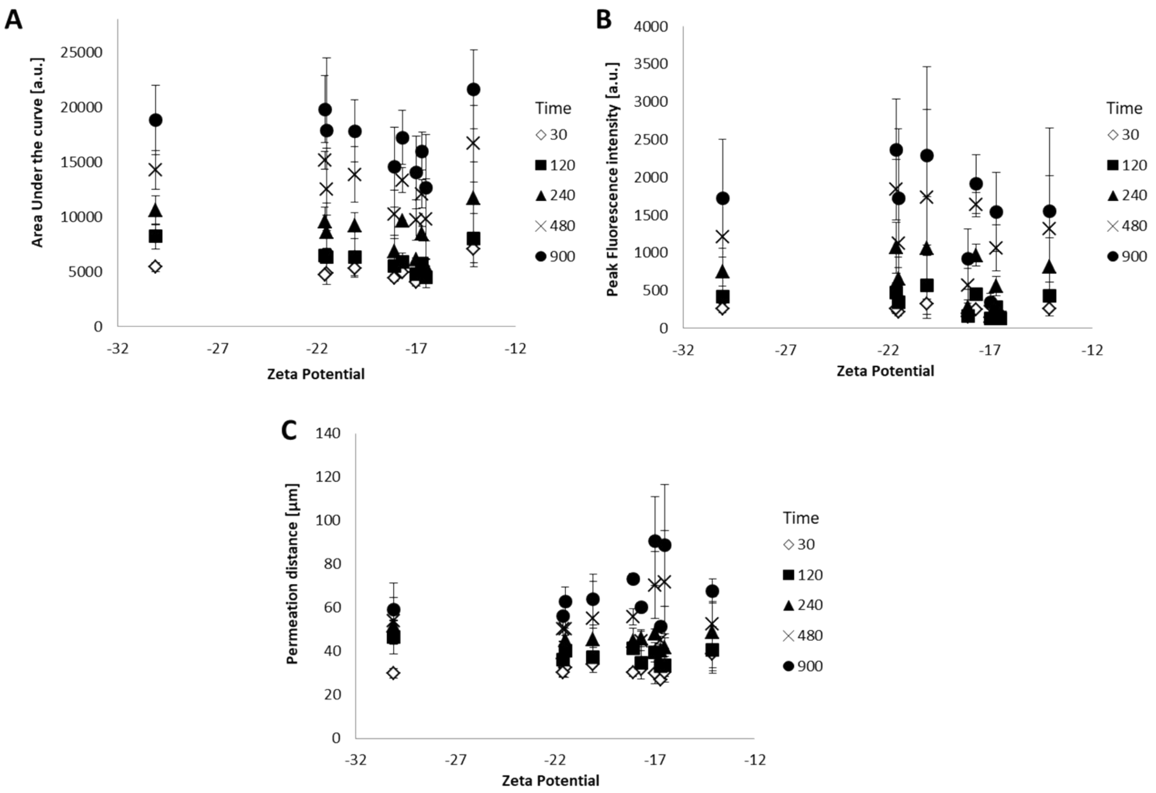

Supplementary Figure 4. Zeta potential of the liposomes does not describe their accumulation in the collagen gel measured as the (a) area under the curve representing total association, (b) peak fluorescence intensity as a measure of accumulation at the interface, and (c) maximum permeation distance.

Supplementary Table 1. Fit of linear regression model between AUC vs time for all liposomal formulations.

Formulation	Rf/D	Average R-squared	Standard deviation R-Squared*
DOPC	0	0.96	0.04
2.5% PEG 1K	0.67	0.95	0.02
5% PEG 1K	0.87	0.96	0.02
10% PEG 1K	1.07	0.95	0.02
2.5% PEG 2K	0.91	0.96	0.05
5% PEG 2K	1.29	0.96	0.03
10% PEG 2K	1.79	0.99	0.00
2.5% PEG 5K	1.78	0.97	0.00
5% PEG 5K	2.02	0.99	0.01
10% PEG 5K	2.74	0.92	0.06

*Standard deviation calculated from 3 replicates.
